# Carotid Sheath Abscess Caused by a Tooth Decay Infection on the Opposite Side

**DOI:** 10.1155/2015/739630

**Published:** 2015-03-23

**Authors:** F. Ruya Tuncturk, Lokman Uzun, M. Tayyar Kalcioglu, Oguz Kadir Egilmez, Emine Timurlenk, Muferet Erguven

**Affiliations:** ^1^Department of Otorhinolaryngology, Medical Faculty, Istanbul Medeniyet University, Goztepe Training and Research Hospital, 34722 Istanbul, Turkey; ^2^Department of Pediatrics, Medical Faculty, Istanbul Medeniyet University, Goztepe Training and Research Hospital, 34722 Istanbul, Turkey

## Abstract

Deep neck infections are mortal diseases that need emergency treatment. It can occur at any age but usually in pediatric ages. In this report, a left cervical carotid space abscess of a pediatric patient was discussed. It was interesting that the only origin of the left carotid sheath abscess was right inferior first molar tooth decay. Right neck spaces were all clean. Patient had no immunosupression and also there were no congenital masses such as branchial cleft cysts, foreign bodies, or masses suspicious for malignancies in cervical ultrasound and MRI. We discussed this rare condition under the light of the literature.

## 1. Introduction

Deep neck infections (DNIs) are infectious process of the spaces between fascias in the head and neck region. They can occur at any age, but pediatric DNIs are much more mortal diseases that need emergency treatment because of their rapidly progressive nature. Characteristic findings include fever, sore throat, dysphagia, dyspnea, and warm, tender neck masses. Oral cavity infections, foreign bodies, traumas, Pott disease, and congenital masses such as branchial cleft cysts, laryngoceles, thymic cyst, branchiogenic cyst, thyroid cyst, thyroiditis, and branchial arch anomalies or masses suspicious for malignancies are the etiologies of deep neck infections [1]. Diabetes, HIV, some medications, malignities, and immunosupressive diseases make the prognosis progressive and fatal. Infections are typically polymicrobial, with* Staphylococcus* and* Streptococcus* being the most commonly isolated pathogens. It is usually seen in the same side of the origin. However, in this paper, we discussed a left cervical carotid sheath abscess of a pediatric patient where the only origin we found was a tooth decay infection on the opposite side.

## 2. Case Report

**Figure 1:**
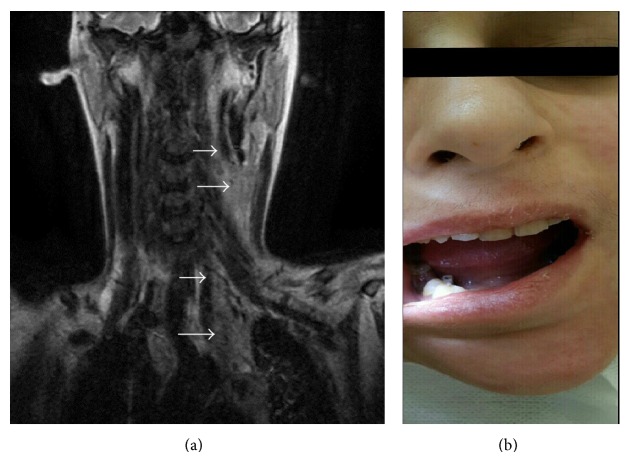
(a) Cervical MRI images (coronal section) that show multiple abscess formation in left carotid space from skull base to the mediastinum and submental lymphadenopathy. (b) The images of the patient after tooth extraction and a Penrose drain application by the consultant dentist.

**Figure 2:**
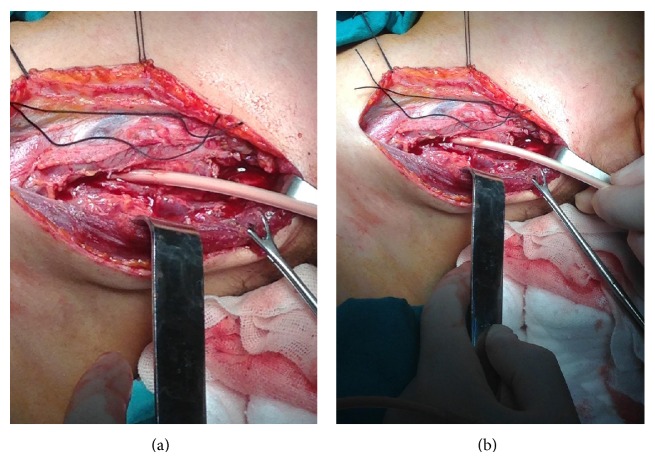
Peroperative images of the patient during the surgical abscess drainage procedure.

A 14-year-old female was referred to emergency service with the symptoms of nausea and headache. In her oropharynx examination, only poor oral hygiene was seen by the pediatrician. There were no infection findings in her ear, nose, and throat. Her chest graphy was normal. Her urine tests and CBC were in normal limits. Analgesics were prescribed and control examination was suggested. After three days toothache started. Her parents took her to a dentist. It was said that there was a tooth abscess around right inferior first molar tooth decay. Amoxicillin and paracetamol were started and control examination was suggested. However, after two days with medication, the patient was getting worse. Submental swelling, dysphagia, and chest pain started. WBC was 20.700 per mm^3^, CRP was 35,7 mg/L, and her fever was 39°C. PA chest graphy was performed and a minimal increase in cardiothoracic index was detected. Neurological examination was normal. There were no pathology about eye movements, muscle strength, and tendon reflexes. Throat and blood cultures and antibiogram were performed. The patient was hospitalized in pediatrics clinic because of a lack of oral intake. Intravenous ceftriaxone and metronidazole medications were started at the first day of hospitalization. As the patient consulted otolaryngologist, in spite of impaired mouth opening abscess formation and purulent secretion from right inferior first molar tooth decay were seen. Impaired mouth opening is more likely because of the ache during the activity of the mandibula, not like trismus. Multiple lymph nodes were palpated in the submental region and bilateral submandibular and anterior cervical regions. There was tenderness in both cervical areas. Consultation by a dentist was suggested. In dental examination, abscess formation, an opening in periodontal tissue, and purulent secretion from right inferior first molar tooth decay were seen. Oral hygiene was poor but no other significant pathology was seen in oral cavity. Tooth extraction was performed and a Penrose drain was applied by the consultant dentist at the third day of the hospitalization in order to avoid chronical process of the infection (Figure 1(b)). The results of the throat, abscess, and blood cultures were negative. There was no bacteria colonisation. However, there was no clinical improvement. In fact the clinic of the patient got worse. Vancomycin was added to the antibiotherapy at the fourth day of the hospitalization, not to ignore the possibility of MRSA infection until evaluating the culture results. Parents of patient denied any systemic disease. Total Ig A, Ig M, Ig G, and Ig E levels were within normal limits. Serologic studies for hepatitis and HIV were negative. Cervical MRI revealed multiple abscess formation in left carotid space from skull base to the level of carina as obliterating the aortopulmonary window in mediastinum and multiple lymphadenopathy in submental, bilateral, submandibular, and anterior cervical areas (Figure 1(a)). Percutaneous drainage under ultrasonography was performed. It was intended to be therapeutic. However, it was not successful because of the dense contents of the abscess. Because of that, in general anesthesia left deep neck spaces were explored. Carotid sheath was opened from the level of carotid bifurcation to the level of clavicula and abscess was drained by an aspirator and a catheter placed in mediastinum through carotid sheath (Figures 2(a)-2(b)). For proximal and distal end, two drains were placed into spaces. Postoperatively place of the drain was checked by PA chest X-ray. Cardiothoracic index was increased and the right costophrenic angle blunting was seen. By echocardiography, pericardial effusion was seen 6.1 mm. There was no cardiac tamponade or any other intracardiac mass. Because of the pleural effusion, chest tube thoracostomy was performed to the right side by the consultant pediatric surgeon. Bacterial cultures of pleural effusion were positive. Proliferation of alpha hemolytic streptococcus was seen and there were only resistances of cefazolin and trimethoprim/sulfamethoxazole in antibiogram. Vancomycine was stopped at the fifteenth day of the hospitalization. For pericardial effusion, clinical followup without treatment was suggested by the consultant pediatric cardiologist. After eight days from the surgery, the general situation of the patient got much better and all drains were removed. WBC was 5.500 per mm^3^, CRP was 2.2 mg/L, and her fever was 36.7°C. After a few days, the periodontal tissue was seen normal. There was no problem about oral intake.

## 3. Discussion

Deep neck space infections are very rare nowadays and it is easy to manage 90% of patients due to the good knowledge of anatomy of three spaces (retropharyngeal, lateral pharyngeal, and submandibular) [2]. The carotid sheath is a tube-shaped fascia that is formed by all three layers of deep cervical fascia and wrapping the common carotid artery, internal carotid artery, internal jugular vein, and 10th cranial nerve [3]. The sheath blends with the thyroid fascia anteromedially and with the deep surface of sternocleidomastoid anterolaterally. It is attached to prevertebral fascia posteriorly along the tips of the transverse processes of vertebrae [3]. It starts from the skull base where it attaches to the jugular foramen and carotid canal. The carotid sheath fuses with scalene fascia, adventitia of great vessels, and the fibrous pericardium inside the mediastinum, inferiorly [3].

As differential diagnosis of the pediatric neck inflammations, bacterial infections (lymphadenitis, deep or superficial abscess, cat-scratch disease, Lemeirre's disease, actinomycosis,* Brucella*, syphilis, tularemia, atypical mycobacterium, and mycobacterium tuberculosis), viral infections (viral adenitis, EBV, HIV, and CMV), fungal infections (histoplasmosis, aspergillosis, toxoplasmosis, and lymphatic filariasis), benign neoplasms (hemangioma and lymphangioma), malign neoplasm (lymphoma and rhabdomyosarcoma), and inflammatory diseases (Kawasaki, sarcoidosis, and Marshall's syndrome) should be kept in mind [4].

Neck space infections are mostly polymicrobial. Recent studies of 64 cases by Dharambir et al. showed Gram-negative organisms from 26 of 34 positive cultures. No bacterial growth was seen in 22 cultures. Using antibiotics before culture sampling and high dose of intravenous antibiotics before surgical drainage may have resulted in negative cultures. Also, in our case, culture was negative for aerobic and anaerobic bacteria probably due to antibiotic therapy started before surgical drainage [2].

Infection in this space usually occurs because of contamination as a result of trauma to the upper aerodigestive tract or secondary to infection from surgical procedures in the neck region. Secondary involvement of the space may occur by extension of infection from lateral pharyngeal space or retropharyngeal space or primary process in the lung or mediastinum [2]. The space can be infected directly by injection of drugs in intravenous drug abusers [2]. If the origin is aerodigestive system, deep neck infections commonly occur on the same side of the origin. In this case, it was interesting that in spite of preoperative investigations and peroperative clinical examinations, there was no other infection origin of the left carotid sheath abscess except for right inferior first molar tooth decay. Patient had no immunosupression and also there was no trauma history or congenital masses, such as branchial cleft cysts, laryngoceles, thymic cyst, thyroid cyst, thyroiditis, and branchial arch anomalies, foreign bodies, masses suspicious for malignancies in cervical ultrasound and MRI. Right neck spaces were all clean [4]. In the literature, there is no case about deep neck infection originated from contralateral tooth abscess as the case discussed in our paper. As a theory, we tried to explain this case by anatomical relations. The submandibular space is located between the mylohyoid muscle and superficial structures within the submandibular triangle. Along the posterior free edge of the mylohyoid muscle, this space is continuous with the submental space within the submental triangle. Although typically infection in this space will not spread in the infrahyoid direction, there is a possibility of the infection to spread from one submandibular space across to the contralateral space [5]. Also, the drainage of the tooth abscess and the Penrose drain may have prevented the ipsilateral spreads.

## 4. Conclusion

While a life-threatening deep neck infection is an uncommon complication of tooth abscess, dentists and otolaryngologist must be able to recognize the signs and symptoms. If no clinical improvement is seen in followups by medical treatment for tooth abscess, the tooth extraction must be performed. These kinds of mortal complications are mostly seen in immunosupressive patients.
